# Advancing Microfluidic Immunity Testing Systems: New Trends for Microbial Pathogen Detection

**DOI:** 10.3390/molecules29143322

**Published:** 2024-07-15

**Authors:** Yiran Wang, Jingwei Chen, Yule Zhang, Zhijin Yang, Kaihuan Zhang, Dawei Zhang, Lulu Zheng

**Affiliations:** 1 Engineering Research Center of Optical Instrument and System, The Ministry of Education, Shanghai Key Laboratory of Modern Optical System, University of Shanghai for Science and Technology, Shanghai 200093, China; 2 2020 X-Lab, Shanghai Institute of Microsystem and Information Technology, Chinese Academy of Sciences, Shanghai 200050, China; 3 Shanghai Engineering Research Center of Environmental Biosafety Instruments and Equipment, University of Shanghai for Science and Technology, Shanghai 200093, China; 4 Shanghai Institute of Intelligent Science and Technology, Tongji University, Shanghai 200092, China

**Keywords:** microbial pathogen, microfluidic, immunoassay, high throughput, rapid diagnosis

## Abstract

Pathogenic microorganisms play a crucial role in the global disease burden due to their ability to cause various diseases and spread through multiple transmission routes. Immunity tests identify antigens related to these pathogens, thereby confirming past infections and monitoring the host’s immune response. Traditional pathogen detection methods, including enzyme-linked immunosorbent assays (ELISAs) and chemiluminescent immunoassays (CLIAs), are often labor-intensive, slow, and reliant on sophisticated equipment and skilled personnel, which can be limiting in resource-poor settings. In contrast, the development of microfluidic technologies presents a promising alternative, offering automation, miniaturization, and cost efficiency. These advanced methods are poised to replace traditional assays by streamlining processes and enabling rapid, high-throughput immunity testing for pathogens. This review highlights the latest advancements in microfluidic systems designed for rapid and high-throughput immunity testing, incorporating immunosensors, single molecule arrays (Simoas), a lateral flow assay (LFA), and smartphone integration. It focuses on key pathogenic microorganisms such as SARS-CoV-2, influenza, and the ZIKA virus (ZIKV). Additionally, the review discusses the challenges, commercialization prospects, and future directions to advance microfluidic systems for infectious disease detection.

## 1. Introduction

Pathogenic microorganisms, encompassing viruses, bacteria, fungi, protozoa, and helminths, are capable of causing diseases and can be transmitted through various means [1,2]. These pathogenic microorganisms, especially SARS-CoV-2, influenza, Zika, and Dengue Fever (DENV), have caused widespread global outbreaks and continue to pose significant public health challenges [3]. The COVID-19 pandemic has affected over 200 million people worldwide, leading to millions of deaths, severe economic downturns, and widespread disruptions to everyday life [4]. The World Health Organization’s data indicate that influenza represents a substantial global health threat, affecting 5% to 15% of the world’s population and causing 250,000 to 500,000 deaths annually [5,6]. Additionally, ZIKV poses a significant concern, particularly in tropical regions with limited resources, and has been designated by the WHO as a global health emergency. On the other hand, pathogenic agents including *Escherichia coli*, *Salmonella*, and *Pseudomonas aeruginosa* can lead to severe clinical outcomes, such as food poisoning, wound infections (especially in burn injuries), and sepsis. Detecting pathogens at the point of need is crucial not only for clinical diagnosis but also for environmental monitoring.

Immunity tests detect antibodies to these pathogens, which not only confirm past pathogen infection in individuals but also aid in tracking the immune response of the host to the pathogen. Therefore, immunity tests have become an important tool for epidemiological research and vaccine development. The common serological testing methods currently used include ELISA, CLIA, etc. [7,8]. However, these existing methods rely on large laboratory equipment, specialized technical personnel, and complex operating procedures, which are time consuming, low throughput, and increasing the cost of testing due to their substantial consumption of samples and reagents. Thus, the automated, miniaturized, and low-cost methods for rapid and high-throughput detection of pathogens are essential in the area of clinical diagnosis and environmental monitoring.

Microfluidics has experienced rapid development since the early 1990s. Microfluidic systems are unique devices designed for automated manipulation, featuring microchannels that facilitate liquid flow within the range of 10–300 μm. These systems incorporate various components and can be integrated with several detection techniques, such as electrochemical, fluorescence, and nanotechnology. Representing a novel generation of traditional detection methods, microfluidic systems streamline processes like specimen collection and preparation, reagent manipulation, bioreaction, and detection, all seamlessly integrated into a single platform. The advantages of employing microfluidic systems in diagnostics include rapid detection, user-friendly operation, cost effectiveness, and precise identification of pathogen-related infectious diseases. Thus, chip-based laboratory serological testing methods provide a more convenient and rapid solution for detecting pathogen-related antibodies [9,10]. Chip-based laboratory assays that integrate traditional laboratory operations onto microchips will become a potential development direction in the future. This paper mainly discusses advancing the immunoassay for microbial pathogen detection through microfluidic system. Additionally, we summarize the sensitivity, specificity, and detection limits of various detected pathogens via different methods (Table 1). This paper provides a unique insight for the application of microfluidic systems to the rapid and high-throughput detection of pathogens.

## 2. Fabrication Methods for Microfluidic Chip in Microbial Pathogen Detection

Traditional detection technologies often rely on large detection equipment and complex operation processes, which are limited in the development of miniaturization and high sensitivity. Microfluidics provides a new solution for related technical breakthroughs: The micrometer scale of the microfluidic platform enhances the scalability of the devices and meets the requirements for sensor and system miniaturization [45]. In addition, the patterned design of microfluidics makes it capable of constructing multi-channel structures, which improves the capability of the multiplexing and high-throughput operation of the detection systems. On the other hand, microfluidics can enhance the selectivity, specificity, and detection time of the system by adjusting the flow rate, channel geometry, etc., which are significant sensing parameters in bio-chemical analysis [46]. Therefore, applicable manufacturing methods are important to the microfluidic systems.

Thus far, there have been a variety of technologies used in the manufacture of microfluidic systems. Among them, soft lithography is a widely used manufacturing technology for producing high-resolution and high-light-transmittance microfluidic chips. By stacking multiple layers, soft lithography enables the fabrication of complex 3D structures. However, high accuracy requires high demands for the mask, which results in expensive manufacturing costs [47]. Carbon dioxide (CO_2_) laser ablation technology has the characteristics of high-efficiency and high-manufacturing accuracy and is suitable for processing polymers. Nonetheless, the equipment is costly, and the operation is complex [48]. Screen printing technology is commonly employed in paper-based microfluidic chips due to its cost effectiveness and flexibility, although system accuracy and stability are insufficient [49]. Furthermore, inkjet printing technology can be utilized to create paper-based microfluidic devices with high throughput and efficiency, but it faces challenges related to the refilling of conductive ink [50]. As an emerging manufacturing method, 3D printing technology can accurately control the assembly of structures to meet the design requirements of complex structures. However, 3D printing technology is difficult to achieve rapid and high-resolution manufacturing. It also requires complex surface treatment processes [51].

## 3. The Application of Microfluidic Chip in Microbial Pathogen Detection

**Figure 1:**
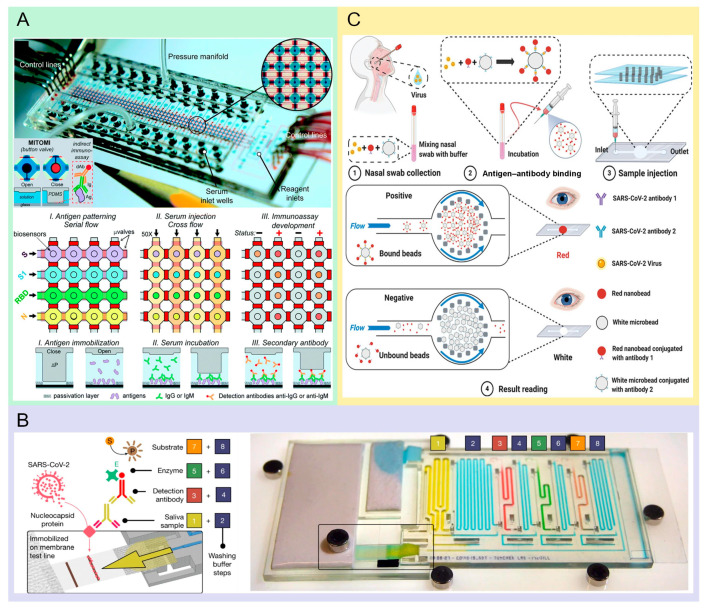
Microfluidic chip in microbial pathogen detection. (**A**) High-throughput microfluidic device in detecting the antibodies of SARS-CoV-2. The figure depicts both the physical layout of the microfluidic chip and the analytical diagram of the microfluidic system. Reproduced with permission [11]. Copyright: The Royal Society of Chemistry of 2021. (**B**) Automated detection of SARS-CoV-2 antibody in saliva was realized by MCR technology. “Microfluidic Chain Reaction” chip in which the sequence of operations described is structurally embedded in the chip design and actuated by capillary action, using a simple piece of paper as a pump. Steps 1, 3, 5, and 7 perform a standard ELISA protocol while steps 2, 4, 6, and 8 are washing steps. Figure (right) shows the chip filled with colored solutions to highlight the different reagents and the washing buffer. The large piece of paper provided on the left is used as the pump. Once started, the different reagents and washing buffer with flow sequentially in the detection chamber, performing all of the operations of an ELISA protocol in an automated way. Reproduced with permission [52]. Copyright: John Wiley and Sons of 2021. (**C**) The illustration of the test procedures of the SARS-CoV-2 virus. Reproduced with permission [21]. Copyright: Springer Nature.

Microfluidic technology enhances the capability for low cost and high-throughput analysis. Currently, microfluidic technologies have paved the way for testing for pathogenic microorganisms, attributed to their fast, economical, and sensitive detection characteristics. Rodriguez-Moncayo et al. have developed a high-throughput device that can evaluate the presence of four distinct SARS-CoV-2 antibodies in as many as 50 serum samples concurrently. The microfluidic system, comprising 200 microchambers, with each chamber holding a volume of 5.5 nanoliters, is controlled via valves equipped with a central button. This design allows for the automated loading of reagents through valve operation and computerization. The sensitivity of this assay is 95%, with a specificity of 91% and a detection limit of 1.6 ng/mL [11] (Figure 1A). Swank et al. explain that it is important to guide public health decisions to establish the true spread of the COVID-19 virus and know how many people have been exposed (including those who only had a mild disease or no symptoms at all). They define a microfluidic immunoassay that can be run in parallel on up to 1024 samples. The testing methodology integrates blood test strips with two proprietary devices, achieving a specificity and sensitivity of 100% and 98%, respectively, with a detection limit of 1 nM, across a pool of 289 human samples. The blood test strips require only a simple finger prick to easily obtain 0.6 mL of whole blood. The microfluidic system uses a standard dual-layer PDMS structure, consisting of a flow and a control layer. The precise management of fluids is executed by manipulating pneumatic valves within the control layer. This technology achieves cost-effective sampling of minute blood through microfluidic nanoscale immunoassays, which are capable of detecting up to 1024 samples per device. These innovative technologies are required to enable the large-scale detection of antibodies specific to the virus. Additionally, these advancements are crucial for monitoring the quality and duration of immune responses [12]. In tackling the task of developing an assay that is both user-friendly and readily deployable for detecting past exposure to and immune reaction against SARS-CoV-2, Heggestad et al. devised a detection method that uses inkjet printing technology to produce stable, spatially discrete capture points for antibody assay reagents, which can evaluate antibody levels in plasma samples from 31 severe COVID-19 patients—41 in good health and 18 infected with the coronavirus. This method is highly sensitive and specific, enabling the tracking of antibody conversion and showing good concordance with live virus neutralization assay results. A completely independent immunoassay platform with minimal user intervention was performed, which greatly simplified the detector design and reading processes. The sensitivity and specificity of this assay both achieved 100% and a detection limit of 0.12 ng/mL [13]. To operate independently from external attachments, devoid of additional components, embed algorithms directly into their structure, and have the potential to become an economical, adaptable, authentic lab-on-a-chip solution applicable across various tasks, including fluid manipulation and on-the-spot medical testing, Yafia et al. [52] proposed the concept of microfluidic chain reaction (MCR). MCR relies on the conditional and structural programming propagation of capillary flow events, achieving precise control over complex processes. This technology was used in the automated detection of SARS-CoV-2 without requiring any external intervention to switch fluids. With parallel operation, timers, synchronous flow cycles, and flow cessation operations, they achieved continuous sampling and analysis of activated clotting plasma and automated detection in saliva. This microfluidic system uses paper as a capillary pump; consequently, there is no need for any complicated actuation system. MCR invented by Yafia at al. [52] is not used by anyone else. The interest of this microfluidic system is demonstrated by performing all the steps of a standard ELISA protocol, and can also be used to perform other bioassays that require sequential fluid manipulation [52] (Figure 1B).

As antibodies produced within the body spend a week to obtain detectable concentrations [33], this implies that serological antibody detection methods cannot provide timely and effective information for detecting the infection of pathogenic microorganisms. Therefore, the identification of viral antigens has emerged as a vital approach in the rapid diagnosis of COVID-19. Tan and collaborators have fabricated a portable microfluidic ELISA platform that identifies anti-SARS-CoV-2 in human serum samples requiring just 8 mL of volume, detecting S1 and N proteins of the virus within 40 min with sensitivity in the picogram per milliliter range (a detection limit of 4 pg/mL) [37]. Furthermore, to achieve rapid, low cost and self-testing, a novel microfluidic system combines a sandwich immunoassay for detecting SARS-CoV-2 proteins. It achieves a rapid (<30 s) separation and enrichment of antigens with a low limit of detection (<100 copies mL^−1^). Clinical trials demonstrate high sensitivities (95.4% and 100%), and the chip’s low cost (USD 0.98/test) and reusability (over 50 times) make it suitable for resource-limited settings [21] (Figure 1C). The microfluidic test kit’s characteristics allow it to exceptionally adapt for regions with limited medical infrastructure and scarce laboratory resources.

Wang et al. reported an immunological microchip method for the rapid detection and quantification of bacteria. This technology uses lipopolysaccharide-binding protein for *E. coli* capture in various mediums, boasting a detection range from 50 to 4000 CFU/mL and proving effective for a spectrum of pathogenic detections in POC settings [42]. Park and colleagues innovated a plastic-based, assembly-free 3D microfluidic magnetic concentrator, advancing pathogen detection. This pre-concentrator selectively pre-concentrates *Escherichia coli* O157:H7 at a 700-fold ratio from 100 mL samples within 1 h and separates and enriches them using antibody-conjugated MNPs. When paired with an ATP luminometer, this concentrator is adept at identifying *Escherichia coli* O157:H7 in blood, even at minimal concentrations of 10 CFU/mL. This pre-concentrator is compatible with multiple detection systems such as ATP luminometer, enabling the preconcentration of *Escherichia coli* from larger volumes down to minuscule quantities, significantly enhancing the sensitivity of pathogen detection [38].

The use of microfluidics in immunoassays has several advantages, such as the precise control of small fluid volumes, as well as reduced reagent and sample consumption, compared to traditional immunoassay methods, which allows them to be highly efficient and cost effective for multiple types of applications. However, microfluidics also has drawbacks including the potential issues with reproducibility, the complexity of chip fabrication, and the need for specialized equipment and expertise.

The introduction of microbeads as solid supports in microfluidic systems can enhance performance by increasing the specific surface area. Moreover, microbeads are easily surface-modified, thereby further expanding the application potential of microbead-based microfluidic systems. These advantages are particularly crucial for the rapid detection of viruses and bacteria. Magnetic nanoparticles (MNPs) are characterized by high surface-to-volume ratio, low detection impedance, and uniform distribution [37]. Microfluidic devices frequently integrate immunomagnetic separation (IMS) to isolate and concentrate pathogens from complex food matrices efficiently. The combination of microbeads and microfluidic chip technology improves the efficiency and throughput of detection in complex samples [53]. This integration allows for multiplexing, reducing sample and reagent consumption while increasing throughput and efficiency. Lin et al. proposed a chip-based, high-throughput sandwich immunoassay reusability strategy, integrating beads labeled with both color and size, along with rolling circle amplification technology (RCA), capable of simultaneously detecting antibodies and inflammatory biomarkers across pg mL^−1^ and μg mL^−1^. These technological advancements greatly enhance the efficiency and accuracy of assessing neutralizing activity in SARS-CoV-2 antibodies [54]. Antibody levels are associated with immune protection. Wu et al. introduced a self-contained, instrument-free microfluidic device that is capable of directly displaying SARS-CoV-2 antibody levels. Magnetic particles are modified with spike protein RBD to ensure specific binding to anti-spike RBD IgG, while polystyrene microparticles (PMPs) are modified with a secondary antibody against human IgG (anti-human IgG), forming MMPs–antibodies–PMPs complexes. Upon loading onto a capillary-driven microfluidic chip, MMPs and MMP–antibody–PMP complexes are automatically separated under the influence of the magnetic separator. Meanwhile, free PMPs continue to flow until they accumulate in the microchannel in front of a particle dam, which features a narrowing nozzle. Consequently, the concentration of anti-spike RBD IgG is quantitatively visualized by the length of the PMP accumulation, eliminating the need for any detection module. The obtained visual quantitative data can be utilized for both sensitive (a detection limit of 13.3 ng/mL in 70 min) and rapid testing (a detection limit of 57.8 ng/mL in 20 min) [14].

**Figure 2:**
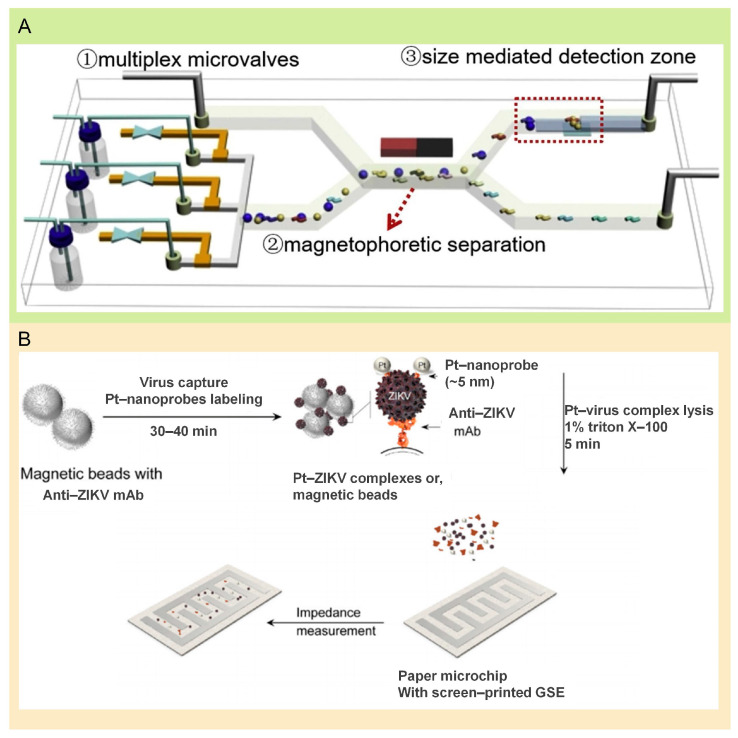
Microfluidic system combined with microbeads. (**A**) Schematic illustration of the microfluidic platform used for detection of H7N9 and H9N2. Microfluidic chip is mainly composed of microvalves, a separation channel, and a detection zone. Reproduced with permission [31]. Copyright: Elsevier B.V. of 2020. (**B**) Structure of the paper-based testing platform fabricated by Draz et al. for the detection of ZIKV. ZIKV envelope monoclonal antibody (Anti-ZIKV mAb)-modified magnetic beads are used to capture virus particles and form complexes with Pt-nanoprobes. Pt-virus complex lysis is detected using a screen-printed graphene-silver electrode (GSE). Reproduced with permission [60]. Copyright: The Royal Society of Chemistry of 2018.

Influenza viruses are classified into types A, B, C, and D, with type A infecting humans and various animals such as pigs and birds, whereas types B and C are typically human-specific. Type A viruses exhibit a diverse array of subtypes, which are differentiated by the structure of their hemagglutinin (HA) and neuraminidase (NA) proteins, numbering up to 144 combinations from HA1 to HA18 and NA1 to NA11 [55,56]. Currently, the influenza virus has been a health concern addressed by “chip laboratories” [28,57,58,59]. In 2020, a team has advanced towards a sophisticated automated microfluidic system focused on H1N1 detection. Their technique utilizes magnetically propelled beads for capturing and identifying antigens, akin to the principles of an ELISA, obtaining a detection sensitivity of 0.032 hemagglutination units per reaction within 40 min [42]. The simultaneous and automated detection of various influenza HA subtypes is essential for early diagnosis and ensuring operator safety. Wang and colleagues have developed a microfluidic system that separates and detects multiplex influenza via size variation in the signal, reaching detection limits of 3.4 nanograms per milliliter for H7N9 HA and 4.5 nanograms per milliliter for H9N2 HA [31] (Figure 2A). The presence of various subtypes poses a significant challenge for detection and treatment, making the simultaneous identification of multiple influenza virus subtypes essential in combating this disease. Hong et al. have introduced a microfluidic chip coded with microbeads, designed for identifying subtypes H1N1, H3N2, and H7N3, according to the magnetic and size differences of the microbeads. This system linearly encoded different subtypes of influenza viruses in different areas of the chip, achieving detection limits of 2.2, 3.4, and 2.9 nanograms per milliliter, respectively [32].

The microbead-based microfluidic chip also was used to detect the ZIKV virus. Chip-based immunoassays are still vital in ZIKV infection detection. Draz et al. developed a microfluidic chip that significantly enhanced sensitivity and specificity by utilizing the platinum nanoparticles of the antibody reaction on the paper-based detection platform. This chip can detect as low as 10 virus particles per μL of ZIKV [60] (Figure 2B). The outbreak of ZIKV highlights the urgent need for affordable clinical diagnostics to accurately identify and differentiate viral infections, thereby enhancing patient care. Given that ZIKV and dengue virus (DENV) are closely related flaviviruses, their similar proteins and nucleic acids can lead to cross-reactions and false positives in molecular, antigenic, and serologic tests. Bosch and collaborators reported a rapid approach for detecting viral NS1 antigens, accurately identifying ZIKV and all four DENV serotypes without any cross-reactivity, with 150 mL serum input, achieving a sensitivity/specificity of 0.81/0.86 for rapid ZIKV detection [33].

Antibody detection, as a common method for detecting bacteria, has seen numerous innovations in traditional detection methods by researchers, driving the continuous development of bacterial antibody detection beyond the combination of microfluidic biosensors and immunomagnetic separation (IMS). Expanding the scope of microfluidic applications, James et al. created a chip that integrates IMS with flow cytometry to enrich and detect Salmonella in food. This system employs a chip equipped with intricate spiral designs to hold immunomagnetic beads, which are later released for flow cytometry-based detection, distinguishing bacterial contamination at levels as low as 10^4^/mL with a magnetic bead recovery rate of 65–85% [61]. Chang et al. introduced a biosensing microfluidic chip embedded with AMP-tagged beads to detect pathogenic *Escherichia coli*. Due to the adoption of a novel channel design, the microfluidic chip achieves reusability and employs inflow-side channels to uniformly distribute bacterial suspensions in the chambers, thereby improving binding efficiency. The LOD for *Escherichia coli* O157:H7 with this method stands at a mere 10 cells/mL, with a detection time within 20 min [62]. Further innovations by Yang et al. include a microfluidic chip specifically for isolating and detecting *E. coli* in urine, particularly for urinary tract infection diagnostics. The LOC consists of tandem dual chambers and an integrated impedance detector. The dual chambers aim to minimize the unintended protein capture that often occurs alongside *E. coli* in urine samples. *Escherichia coli* K-12 is placed in the concentration chamber containing anti-E antibodies conjugated to microscale magnetic beads, and then the immobilized *E. coli* is transferred to a chamber for measuring electrical impedance with the measured impedance change in the sensing chamber displayed as ~60 kΩ. The device detects *Escherichia coli* with a sensitivity of at least 3.4 × 10^4^ CFU/mL [39]. Bloodstream infections (BSIs), which occur when pathogens are present in the blood, represent a significant global health burden, especially when they progress to sepsis and septic shock. Conventional diagnostic methods are often time consuming, lack specificity, or are affected by blood components, hindering the timely and effective treatment of BSIs. Costa and associates have introduced an innovative microfluidic approach using head-based chips and receptor-binding proteins (RBPs) for the precise and sensitive detection of multiple bacteria, including *E. coli* and *Pseudomonas aeruginosa*. The device includes a microcolumn with agarose beads functionalized with antibodies, allowing the capture and separation of target bacteria from blood. This detection method does not need the laborious pre-enrichment steps traditionally required for bacterial identification and can highly specifically detect *Escherichia coli* and *Pseudomonas aeruginosa* in whole blood within 70 min, with an LOD of approximately 10^3^ CFU [44]. The industrialization of microbead-based detection methods combined to microfluidics is progressing rapidly, with increasing adoption in biomedical diagnostics, environmental monitoring, and pharmaceutical research. Numerous companies are developing and commercializing integrated platforms, offering high-throughput and cost-effective solutions for various applications [63,64]. Continuous advancements in materials, manufacturing processes, and automation are driving further growth and innovation in this field.

## 4. Immunosensor-Based Microfluidic Chip

Microfluidic chips facilitate the quick processing of samples, and, when combined with biosensors, they offer rapid detection and analysis, crucial for timely medical decision-making. 

As an efficient and highly sensitive analytical device, biosensors can transform biological responses into detectable electrical signals [65,66]. Biosensors consist of two main elements: bioreceptors, which recognize the target analyte, and transducers, which convert the process of a biological reaction into an electrical signal. Common transducing elements include electrochemical, optical, magnetic, acoustic, and various other methods [67].

Currently, a variety of materials are used to manufacture microfluidic detection platforms with integrated biosensors. Materials play a very important role in implementing high sensitivity of detection, sample preparation, etc. Magnetic materials can enhance biosensing and can be used to separate analytes from samples [68]. Metal materials are good in improving the performance of sensor probes. Additionally, using nanomaterials as the recognition layer can increase the specific surface area of the system, thereby enhancing probe sensitivity [69].

Due to the specificity of antibody–antigen interactions, antibodies have garnered significant attention in biosensors. The immunosensor is a type of biosensor based on solid-state affinity, where the target analyte antigen (Ag) is detected by forming a stable complex with the antibody (Ab) acting as a capturing agent. This immune response causes the sensor to generate a measurable signal [70]. Electrochemical sensors are the most commonly used method for immunosensors [71]. Antigen specificity is identified by trapping antibodies on the surface of the fixed electrode. The level of the analyst is measured by monitoring changes in the current or impedance resulting from the immune response [72].

**Figure 3:**
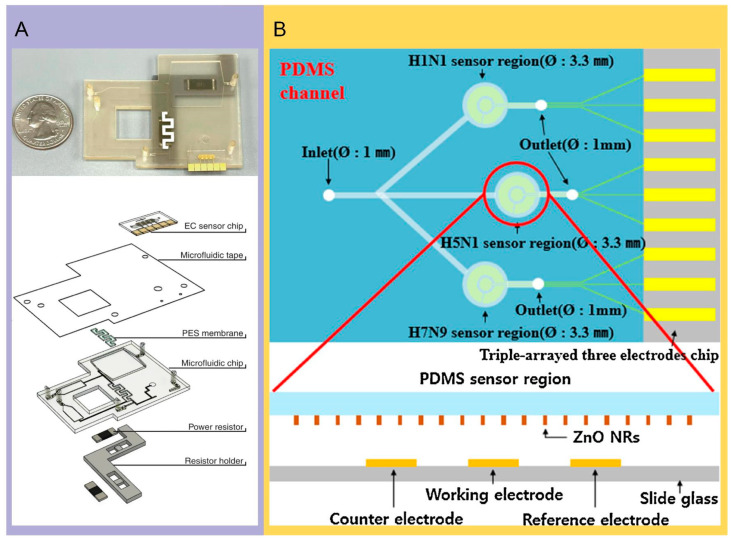
Application of electrochemical technique in microfluidic systems. (**A**) Multichannel electrochemical sensor microfluidic system for detecting SARS-CoV-2 antibody. The exploded view of the microfluidic system mainly includes the sensor chip, membrane, resistor, and microfluidic chip. Reproduced with permission [65]. Copyright: Springer Nature of 2022. (**B**) The structure of the microfluidic electrochemical chip fabricated by Han et al. for multiplexed detection of type A influenza, which consists of three sensor regions for the detection of three strains of influenza A. The cross section shows the relative positions of the counter electrode, working electrode, and reference electrode, respectively. Reproduced with permission [30]. Copyright: Elsevier B.V. of 2015.

**Figure 4:**
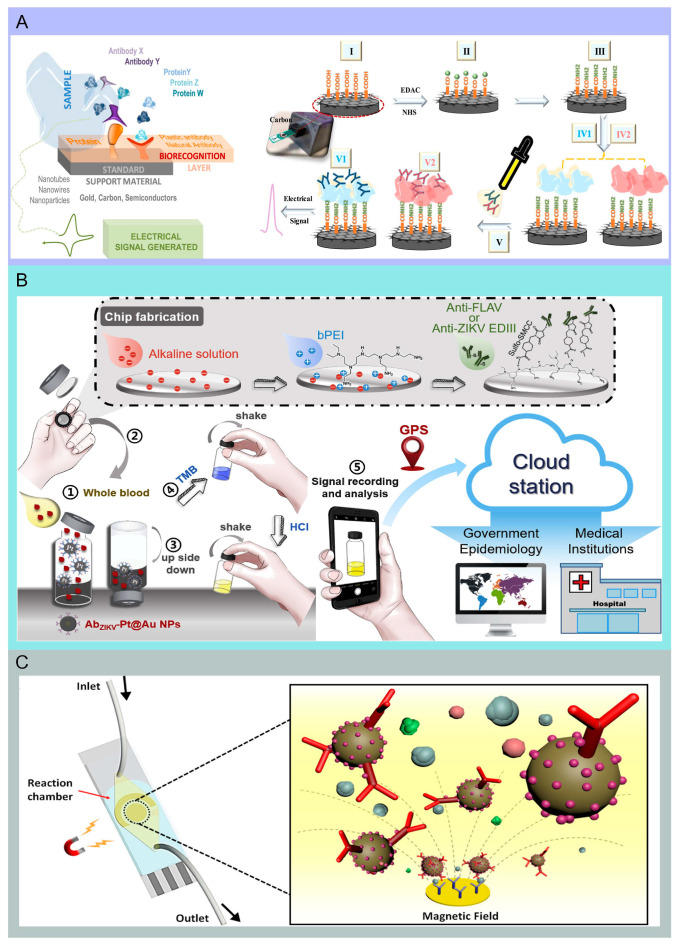
The testing method of biosensors combined with microfluidic platforms. (**A**) Graphical illustration of the immunoassay in microfluidic chip fabricated by Gustavo et al. used for detecting ZIKV antibodies. After oxidizing the carbon material (I), the surface is modified to generate an amine layer (II). Diamine compounds are subsequently added to aminate the surface (III) for accommodating the protein antigens (IV). Eventually, immune antibodies are captured by the sensing layer and generate electrical signals (V). Reproduced with permission [67]. Copyright: Elsevier B.V. of 2018. (**B**) The illustration of manufacturing and testing procedures. Reproduced with permission [34]. (**C**) Schematic diagram of the immunosensor microfluidic system designed by Jiran et al. for SARS-CoV-2 antigen testing. There is a reaction chamber integrated with an immunosensor using immunomagnetic beads for enrichment, between the inlet and outlet in the microfluidic chip. Re-produced with permission [22]. Copyright: American Chemical Society of 2021. Copyright: Elsevier B.V. of 2019.

Electrochemical techniques provide a multiplexed fluidic–impedimetric readout and high sensitivity for detecting low concentration samples. Integrating electrochemical sensing elements into microfluidic channels enhances the detection sensitivity, as well as shortens the analysis time, enabling real-time monitoring [73]. Moakhar et al. have demonstrated a cost-effective multi-channel fluidic device that incorporates a gold nano/micro-islands sensor within the electrochemical biosensor. The system consists of multi-channel fluidic impedance readers and sample preparation modules, enabling data analysis through smartphones. For the 44 COVID-positive and 25 COVID-negative saliva and blood samples tested, this method is shown to be on par with a real-time quantitative polymerase chain reaction. It reports 100% sensitivity and 100% specificity, with a detection limit of 3.13 ng/mL within 11 min [17]. The necessity for a detection method that can rapidly measure antibodies triggered by a SARS-CoV-2 infection is urgent, particularly in regions with strained healthcare resources. A detection scheme that can be read via smartphones would be more desirable and hold greater practical value. Ali and his colleagues have reported that they achieved high sensitivity in detecting S1 and RBD antibodies by utilizing 3D electrodes. The immunosensor is formed by coating the electrode with reduced graphene oxide nanosheets and fixing the viral antigen. This electrode could be integrated with a microfluidic system and then combined with a smartphone-assisted electrochemical readout, with LODs of two types of SARS-CoV-2 antibodies reaching 2.8 × 10^−15^ and 16.9 × 10^−15^ mol/L, respectively [15]. These low-cost, real-time microfluidic sensors offer the potential for the widespread monitoring of COVID-19 infections and immune status. An alternative assay reported by Samper et al., the electrochemical capillary flow immunoassay, effectively quantifies IgG antibodies targeting the virus’s nucleocapsid proteins in blood samples using screen-printed carbon electrodes. This assay reaches detection levels as low as 5 ng/mL within 20 min. Integrating this assay with a smartphone-enabled near-field communication system demonstrates its capability for rapid diagnostics [16]. Co-detection of an RNA and serological antibody response to SARS-CoV-2 aids in determining the immune status of COVID-19 patients. Najjar indicated that microfluidic chips fabricated by means of 3D printing technology can detect RNA in saliva and antibodies in saliva or plasma within 2 h through multiple electrochemical outputs [65] (Figure 3A). They fabricated an electrochemical immunosensor composed of bovine serum albumin and reduced graphene crosslinked with glutaraldehyde (BSA/rGOx/GA). Han et al. introduced a microfluidic electrochemical setup for the multiplexed detection of type A influenza, combining three electrodes with ZnO nanorods (NRs) arrayed on PDMS to create a sensor capable of recognizing multiple strains like H1N1, H5N1, and H7N9, with detection ranging from 1 picogram to 10 nanograms per milliliter. A low limit was 1 pg/mL of each virus [30] (Figure 3B). Since both ZIKV and DENV viruses are transmitted by the same mosquito species, their geographical distributions significantly overlap. Therefore, achieving a reliable serological distinction between these viruses is crucial. Gustavo and team engineered an innovative biosensor due to recombinant ZIKV non-structural protein 1 (NS1) and the domain III of the envelope protein (EDIII), which involves applying two different antigens onto a conductive carbon surface. This biosensor quickly distinguishes between ZIKV and dengue virus-specific antibodies by using techniques such as Electrochemical Impedance Spectroscopy (EIS) and Square Wave Voltammetry (SWV). Capable of detecting ZIKV antibodies in untreated blood and saliva, this biosensor’s performance remains stable even in the presence of antibodies specific to the DENV. It demonstrated sensitivity and specificity ranges of 0.76 to 1.00 for DENV1-4 serotypes and the collective DENV assay. For the rapid ZIKV test, the sensitivity was 0.81 with a specificity of 0.86 [67] (Figure 4A). The combination of microfluidics and electrochemical techniques facilitates the development of highly integrated and automated systems, improving detection efficiency and accuracy while minimizing human error. While there are some commercial applications, such as portable glucose meters and environmental sensors, the overall commercialization of these combined technologies is still in its early stages, with significant potential for future growth.

The optical immunosensor system consists of a light source, a sensor, and a photodetector to analyze antigen–antibody interactions by detecting changes in optical signals within the system [71]. Parallel multi-channel virus antigen analysis is a rapid and powerful integrated all-in-one POCT device designed for multiplex analysis. Teixeira at al. employs functionalized polycarbonate disc-shaped surfaces equipped with microfluidic structures, combined with a portable photonic analyzer, to immobilize specific biological reagents in microarray formats. This device can process 30 mL samples within 30 min, quantitatively detecting the concentration of virus antigens and specific concentrations of immunoglobulin G and M with biosensors. They performed an assay with 135 serum samples as well as 147 nasopharyngeal samples. The results demonstrated that an excellent agreement (0.937) with the detection limit of 17 ng/mL was obtained compared with commercial immunoassays [23]. Lee et al. developed an immunosensor based on the microfluidic detection of H1N1, by modifying the surface of the detection channel with nano-gold particles and fixing the hemagglutinin antigen on the gold surface using genetically engineered peptides, greatly enhancing the detection efficiency and test accuracy [74]. An early, accurate, and specific POC diagnosis of ZIKV presents significant challenges because the majority of infected individuals are either asymptomatic or exhibit nonspecific symptoms resembling those of other viral infections. Furthermore, most diagnostic methods require the use of specialized instruments. Hsu and associates devised a POC method using artificial nanozyme platinum/gold core–shell nanoparticles (Pt@Au NPs) as an immunosensor with a smartphone for ZIKV detection. This method is free from instrumental constraints, exhibiting remarkable sensitivity down to 1 pg/mL of the ZIKV [34] (Figure 4B). Antibodies, proteins generated by the immune defenses of eukaryotes, are crucial in biosensor technology due to their specific affinity for target antigens, making them optimal for bacterial detection in microfluidic platforms. In microfluidic biosensors, these antibodies are commonly fixed onto electrode arrays to spot bacterial presence. Moreover, Park et al. have engineered a rapid, sensitive chemiluminescent enzyme-linked immunosorbent assay for *E. coli* O157:H7 detection, optimizing buffer composition to curtail non-specific protein attachment on the membrane strip and enhancing detection capabilities, resulting in the optimal performance of the immunosensor. A chemiluminescent assay has the capability to quantitatively identify *E. coli* O157:H7, with the concentration detection range from 1.1 × 10^3^ to 1.1 × 10^7^ CFU/mL within 16 min [40]. The ability of antimicrobial peptides (AMPs) to effectively bind to a variety of target microbes has garnered significant attention as a promising alternative to antibodies for whole-bacteria detection. 

Additionally, magnetic-based immunosensors are employed for disease surveillance within microfluidic systems. Jiran and colleagues have designed a novel microfluidic magnetic immunosensor that uses dual-labeled magnetic nanoparticles for both immunomagnetic enrichment and signal enhancement. This innovative method facilitates the testing for SARS-CoV-2 antigen, achieving sensitivities of 50 pg/mL in undiluted serum and 10 pg/mL in serum diluted five times. Additionally, they have employed a mobile smartphone-operated diagnostic tool capable of identifying the nucleocapsid protein of SARS-CoV-2 in serum specimens [22] (Figure 4C). 

Microfluidic devices integrated with biosensors significantly improved detection time and LOD. However, due to the existence of the multiple subtypes of pathogenic microorganisms, the multiplex detection capability of microfluidic chips still needs prompts. Additionally, the continuous evolution and mutation have increased the challenges of this work. Thus, further improvement in the portability and cost-effectiveness of microfluidic chips is needed to make them more practical and feasible. 

In addition, although the integration of biosensors and microfluidic chips is still emerging, there are already commercialized products available, such as glucose monitors and lab-on-a-chip devices, indicating significant potential for future market expansion. However, there remain challenges in the commercialization of microfluidic-based biosensor systems. Currently, achieving modularization and standardization of the components in different systems is difficult, which restricts the widespread application of biosensors in clinical application. Furthermore, advanced signal amplification strategies are still required to enhance sensor sensitivity. On the other hand, developing automated microfluidic inspection systems is crucial to shorten the time-to-answer process, which is important for the commercialization.

## 5. Single Molecule Arrays

Single molecule array (Simoa) technology is an ultrasensitive assay method that amplifies single molecules with an immunoassay that uses antibody-coated beads for detection, enabling precise quantification of low-abundance biomolecules in complex samples [75]. It utilizes microfluidics, fluorescent labels, and a digital readout to achieve unprecedented levels of sensitivity and accuracy in biomolecular analysis [76]. These technologies possess a sensitivity approximately 50 times greater than traditional competitive ELISA methods [77].

A high-sensitivity detection method of antibodies for SARS-CoV-2 is essential to prevent transmission and offering patients with timely care. When SARS-CoV-2 enters the body, its spike protein binds to the angiotensin-converting enzyme 2 (ACE2) receptor present on the surface of cells in the respiratory tract, particularly in the lungs [78]. Gilboa et al. have developed an antibody-mediated ACE2-spike interaction blocking detection method using Simoa [79]. This technique not only displays high sensitivity and the ability to perform multiple tests simultaneously but also eliminates the requirement for live virus or cell cultures. Capable of completing the process within two hours in a level 2 biosafety lab, this detection method has a high sensitivity and excellent detection limit. Furthermore, Thomas et al. utilized Simoa technology to successfully demonstrate the feasibility of diagnosing SARS-CoV-2 patients by detecting IgG and IgA antibodies in saliva. Compared to the traditional ELISA method, the system showed a sensitivity increased by five orders of magnitude, with diagnostic accuracy reaching 100% [80]. On the other hand, Song et al. applied Simoa technology to bacterial detection. Through the detection of labeled compounds, the system effectively identified Staphylococcus aureus, demonstrating the clinical application value of Simoa technology in diagnosis and pathogen detection [81].

In terms of commercial applications, Simoa technology has made significant strides in the early detection of pathogenic microorganism, gaining widespread recognition [82,83]. Numerous biotechnology companies and medical institutions are actively adopting Simoa technology to enhance diagnostic accuracy and efficiency [84]. However, Simoa is associated with high costs for equipment and reagents, and the operation of Simoa requires specialized training and maintenance, increasing the complexity and operational costs. These disadvantages limit its accessibility in resource-constrained laboratories and medical facilities [85].

## 6. Lateral Flow Assay

**Figure 5:**
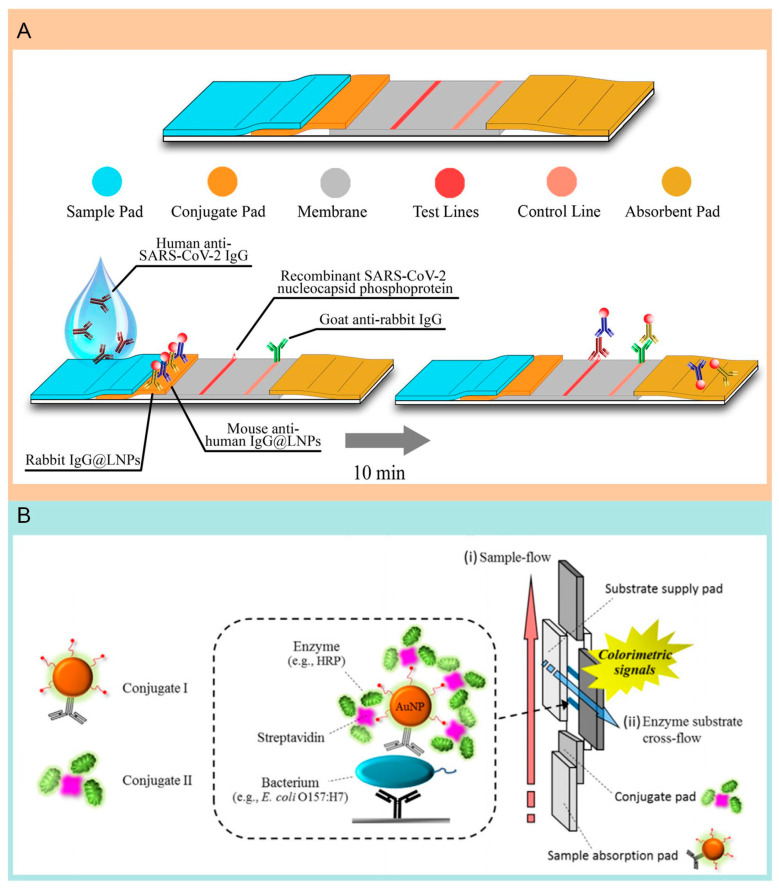
The application of the LFA technique in the detection of pathogenic microorganisms. (**A**) Illustration of the design and fabrication of the LFA assay. Reproduced with permission [70]. Copyright: American Chemical Society of 2020. (**B**) Illustration of signal amplification based on a gold nanoparticle for pathogen detection [43]. Copyright: Elsevier B.V. of 2015.

Lateral flow assay (LFA) technology enables the rapid and simple detection of various biomarkers, meeting the demand for POCT, the low production cost of LFA test strips, and the lack of need for complex instrumentation, which reduce overall testing expenses, offering significant advantages for widespread deployment, especially in resource-limited settings and emergency situations. LFA can provide results in a short time, typically within minutes to half an hour, which is crucial in emergencies, such as during infectious disease outbreaks for rapid screening. The present report demonstrated that, with an LFA using nanoparticles, serum samples could be detected within 10 min [86] (Figure 5A). Similarly, with the advanced materials, Feng et al. conducted research on solid-phase immunoassays using lanthanide europium (III) fluorescence microspheres, a method capable of qualitatively or semi-quantitatively detecting IgG within 10 min. In total, 28 individuals affected with COVID-19 and 77 health individuals were included. The analytical data indicate that the sensitivity and specificity rates for this method are 98.72% for IgG (with perfect 100% specificity) and 98.68% for IgM (93.10% specificity) [26]. Creating rapid, ultra-sensitive, precise, and adaptable instruments to track overall antibody levels within communities has emerged as a pressing necessity. Zhou et al. employed an innovative approach by embedding a huge amount of quantum dots into a matrix to prepare high-brightness quantum dot nanobeads (QBs) and applied them as signal amplification labels in LFA. Compared to conventional LFA methods that utilize gold nanoparticles, this innovative approach marks a significant advance, enhancing sensitivity ten-fold within a mere 15 min [87]. Additionally, to obtain the quantitative and sensitive multiplex detection on the rapid and low-cost system, Chen et al. have introduced a highly sensitive LFIA strip, utilizing Surface-enhanced Raman scattering (SERS) designed for the simultaneous identification of both IgM and IgG antibodies against SARS-CoV-2. This method utilizes gap-enhanced Raman nanotags, where a “hot spot” with a gap of only 1 nanometer between the core and shell enhances the signal approximately 30-fold compared to traditional nanotags. Impressively, the detection limits for IgM and IgG have been brought down to 1 ng/mL and 0.1 ng/mL, respectively, which represents a hundred-fold reduction compared to traditional LFIA strips [19]. 

LFAs have been recognized for their utility in identifying SARS-CoV-2 antigens. In 2022, Peng et al. fabricated an (Lateral Flow Immunoassay) LFIA method using colloidal gold nanoparticles, specifically designed for rapid detection of antigen. This method significantly improves detection sensitivity through a copper deposition-induced signal amplification mechanism, resulting in a three-order-of-magnitude increase in LOD to 10 pg/mL by means of signal amplification processing [27]. Wang and associates also have developed an advanced dual-modal LFA biosensor. This biosensor utilizes magnetic quantum dots for heightened sensitivity, effectively detecting both spike (S) and nucleocapsid (NP) proteins of the virus at detection limits of 1 pg/mL and 0.5 pg/mL, respectively [26]. This method indicated a significant accuracy and specificity in sample detection, which is an efficient system to meet the demand of the antigen tests.

The ability to quickly and sensitively detect bacteria on-site, without the need for advanced equipment or trained personnel, is crucial in clinical environments, emergency response situations, and resource-limited settings. Hossain and team have invented an innovative, ultra-sensitive paper-based assay that utilizes intracellular enzyme activity for multiplexed *E. coli* detection. This assay can distinguish *E. coli* O157:H7 without necessitating cell culture, offering limits of detection as precise as 5 CFU/mL and 20 CFU/mL for *E. coli* BL21. Additionally, with the inclusion of a culture step, bacteria present at less than 1 CFU in 100 mL water samples can be detected within 8 h [40]. Most lateral flow immunochromatographic assays (LF-ICAs) for pathogen detection rely on producing color signals from gold nanoparticle (AuNP) tracers that are visible to the naked eye. However, these methods often exhibit lower sensitivity compared to traditional enzyme-based immunoassays or the levels required by regulatory standards. Cho et al. developed an enhanced LF-ICA system in which many enzymes in the gold nanoparticle AuNP-affinity biotin construct can be labeled to measure very low levels of bacteria. Using this method, it’s possible to detect the bacteria in quantities as low as 100 CFU/mL, indicating that this signal amplification method can increase the LOD by approximately 1000 times [43] (Figure 5B).

Currently, LFA technology is widely commercialized, with numerous products on the market, such as pregnancy test kits and COVID-19 rapid tests. Despite this, there is still significant potential for further development and market growth as technology advances and demand increases.

## 7. Smartphones Integrated with Microfluidic System

**Figure 6:**
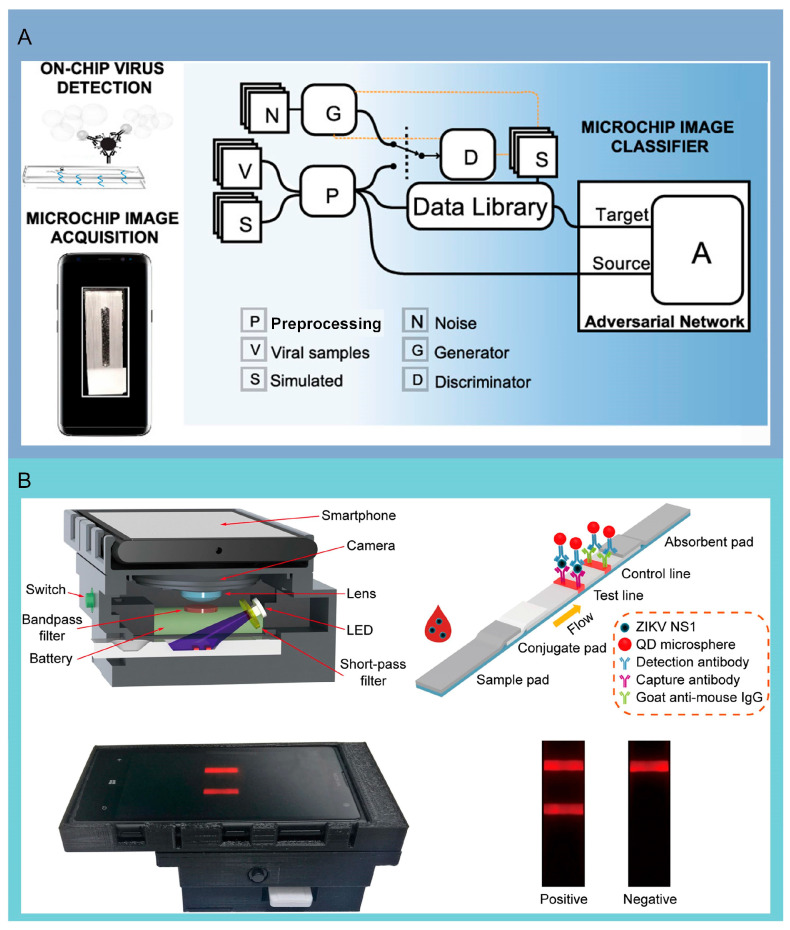
Microfluidic detection platform combined with smartphone. (**A**) Graphical illustration of the general architecture of the adversarial neural networks as defined by Shokr et al. used to analyze the test results captured by the smartphone for SARS-CoV-2 antigen detection. Reproduced with permission [25]. Copyright: American Chemical Society of 2020. (**B**) Overview of the design of the smartphone-based detection system. The detection platform is mainly composed of smartphone, camera, lens, and filter. Reproduced with permission [35]. Copyright: Elsevier B.V. of 2018.

Smartphones can process and analyze data in real time, providing immediate results and enabling timely medical interventions and decision-making, which are widely available and user-friendly, making them ideal for integrating with microfluidic chips to create accessible diagnostic tools for a broader population. The combination of image processing technology using deep learning (DL) with smartphones has been applied to the detection of novel coronavirus antigens. Shokr et al. developed a virus diagnostic platform using conditional generative adversarial networks that are easy to reconstruct. This method can rapidly produce image classifiers for various pathogens by examining data from microfluidic chip imagery captured via smartphones. Verification trials using this platform on 179 patient samples, targeting five distinct antigens, have validated its versatility. Impressively, it has proven a 100% success rate in the detection of SARS-CoV-2 antigens from 62 nasal swab specimens [25] (Figure 6A). Moreover, recognizing the potential for SARS-CoV-2 transmission via bioaerosols, Kim and colleagues have designed a portable, cost-effective, paper-based microfluidic detection system that interfaces with smartphones. This device is adept at directly detecting the virus in airborne droplets or aerosols, eliminating the need for air sampling or extensive sample collection, with the total detection process taking under 30 min. The versatility of these microfluidic devices not only provides convenience for detecting SARS-CoV-2 but also paves the way for other pathogens analyses [88]. Further advancing the field, Rong’s group created a paper-based microfluidic platform integrating quantum dot probes with smartphone interfaces for output reading. This system stands out for its minimal cross-reactivity with other viruses, and it can precisely detect ZIKV in serum samples in just 20 min, with an impressive detection threshold of 0.15 ng/mL [35] (Figure 6B). Smartphones offer robust connectivity options, allowing for seamless data sharing and remote monitoring by healthcare professionals, enhancing patient care and follow-up. While the integration of smartphones with microfluidic chips is still developing, there are already some commercial products and research prototypes available, showing the potential for significant market growth and innovation in the near future.

## 8. Challenges in the Design and Optimization of Immunoassays in Microfluidic System

The format of the conventional sandwich/capture-based immunoassay consists of a capture antibody, sample, and detection antibody. Stable and specific antibodies can not only produce highly sensitive testing results but also reduce cross-reactivity to decrease the detection limits of the microfluidic immunoassay system [89]. Therefore, in the design and optimization of the microfluidic immunoassays, it is particularly important to verify the specificity of the selected antibodies [90]. On the other hand, the low cost and ease of the manufacturing of microfluidic systems enable optimization through chip-based methods, which involve adjusting dilution ratios, incubation times, and washing steps. Kihwan et al. illustrated a microfluidic platform used for the three-level full factorial design of experiments [91]. By changing the analyte concentration and incubation time, the detection limit of the system was reduced by 5 times. Furthermore, the sensitivity and efficiency of microfluidic immunoassays can also be improved by integrating microfluidic modules with hybrid functions [92].

On the other hand, stricter quality assurance and control processes need to be established before the clinical application and broader adoption of the microfluidic immunoassay platform. The calibration curve of the system is affected by the curve-fitting procedures, coefficient of variation (CV), recovery rate, and limit of quantitation [93]. Therefore, to ensure the reliability and accuracy of immunoassay results, biological samples should be used instead of buffer solutions, which lack endogenous markers and cannot objectively reflect the assay performance, during the design stage of the microfluidic systems [94]. The selection of constituent markers should be according to the panel based on circulating protein abundance to ensure that the process meets the quality control specifications [95]. Furthermore, the selection of quality control materials containing known analyte concentrations is important for the continuous monitoring of microfluidic systems to verify their performance. Currently, the exclusion of outliers to ensure quality control mainly relies on the Levey-Jennings charts, which necessitate a complex calculation of the representative indicators of each standard curve [96]. Therefore, developing better statistical methods and quality control algorithms is particularly significant for improving the CV of the system [97].

The standardization of procedures and results is closely linked to biomarker validation and quality control. The establishment of a set of biomarker panels suitable for common conditions not only facilitates comparisons between different microfluidic testing systems but also enables users to select personalized and accurate platforms in clinical practice [98]. Standardized strategies can be achieved through the strict control of the immunoassay design and standardization of the experimental process. Furthermore, the standardization of data reporting and the development of common experimental equipment are driving progress in the comparison of the efficiency between different studies and technologies [99]. 

In addition to optimizing the immunoassay performance, integrating advanced computational methods, such as machine learning, and developing automated data processing and analysis modules to replace the labor-intensive detection process can enhance the application and impact of the microfluidic system. In machine learning, complex statistical models are trained for pattern recognition and to predict events [100]. Currently, researchers have developed various clustering modes and supervised classification algorithms, such as neural networks and support vector machines [101]. However, due to the differentiation of different microfluidic systems, machine learning models often perform well only on trained data, posing a risk of overfitting and limiting the construction of cross-platform data [102]. Moreover, the complexity of fluid dynamics exacerbates this effect, leading to significant performance differences in microfluidic systems across various fluid environments. Hence, a variety of analytical methods should be combined to verify the accuracy of pattern recognition algorithms, ensuring compliance with appropriate regulations such as Food and Drug Administration approval before further application [103].

## 9. Conclusions

This review article focuses on the diagnostic methods based on microfluidic systems that have emerged for immunity tests to detect pathogenic microorganisms, especially SARS-CoV-2, influenza virus, ZIKV, etc. Firstly, the manufacturing methods of the microfluidic platforms are discussed and compared. Considering the production cost and biocompatibility, it is particularly important to select a suitable preparation method for microfluidic systems. Compared to traditional testing methods, which require complex processes and are time consuming and expensive, microfluidic systems offer advantages such as lower costs, improved speed, reduced sample consumption, and greater simplicity. Therefore, immunity testing based on microfluidic systems holds significant value in detecting microbial pathogens. This review also introduces microfluidic systems integrated with other technologies for pathogenic microorganism detection. Microfluidic systems with integrated biosensors have been widely used. Immunosensors, which depend on the specific binding of antibodies, have shown significant advancements in the fields of electrochemistry, optics, and magnetism. Furthermore, the development of microfluidic systems has spurred advancements in Simoa and LFA technologies. We also discussed the research on combining microfluidic platforms with smartphones. These technologies enable the quick and easy testing of pathogenic microbial signals via smartphone devices. Additionally, we have discussed the design and optimization of immunoassays in microfluidic systems. Enhancing quality assurance and control, developing standardized preparation protocols, and selecting appropriate pattern recognition strategies effectively improve the efficiency of immune response.

In summary, microfluidic systems provide rapid, high-throughput, and cost-effective platforms for the accurate testing of pathogenic microorganisms. This has significantly expanded their application in the biomedical field, particularly in the diagnosis of infectious diseases. However, the current design of the microfluidic detection system still has several limitations. Flexible assembly of microfluidic chips might attract the wide attention of researchers. Meanwhile, most of the existing microfluidic systems rely on supporting detection equipment, which greatly limits the actual promotion and application. The integrated, modular, and portable microfluidic platform may become a research interest with great development space. Therefore, by integrating diverse detection methodologies, rapid, high-throughput, flexible, and miniaturized microfluidic platforms are set to become a groundbreaking instrument for conducting immunity tests against a range of pathogenic microorganisms. For the next generation of microfluidic systems for the detection of pathogenic microorganisms, speed, efficiency, and connectivity are gradually becoming critical technical aspects.

## Figures and Tables

**Table 1:** List of various pathogens detected by microfluidic systems with sensitivity, specificity, and detection limit.

Pathogenic Microorganisms	Detection Limit	Sensitivity	Selectivity	Ref.
SARS-CoV-2	1.6 ng/mL	95	91	[11]
SARS-CoV-2	0.12 ng/mL	100	100	[12]
SARS-CoV-2	1 nM	98	100	[13]
SARS-CoV-2	13.3 ng/mL	-	-	[14]
SARS-CoV-2	2.8 × 10^−15^ M	-	-	[15]
SARS-CoV-2	5 ng/mL	-	-	[16]
SARS-CoV-2	3.13 ng/mL	100	100	[17]
SARS-CoV-2	-	98	100	[18]
SARS-CoV-2	0.1 ng/mL	-	-	[19]
SARS-CoV-2	-	99	99	[20]
SARS-CoV-2	-	95	100	[21]
SARS-CoV-2	230 pg/mL	-	-	[22]
SARS-CoV-2	17 ng/mL	100	98	[23]
SARS-CoV-2	4 pg/mL	-	-	[24]
SARS-CoV-2	-	100	100	[25]
SARS-CoV-2	0.5 pg/mL	-	-	[26]
SARS-CoV-2	10 pg/mL	-	-	[27]
H1N1	0.5 PFU/mL	-	-	[28]
H1N1	0.032 HAU	-	-	[29]
H1N1	1 pg/mL	-	-	[30]
H5N1	1 pg/mL	-	-	[30]
H7N9	1 pg/mL	-	-	[30]
H7N9	3.4 ng/mL	-	-	[31]
H9N2	4.5 ng/mL	-	-	[31]
H1N1	2.2 ng/mL	-	-	[32]
H3N2	3.4 ng/mL	-	-	[32]
H7N3	2.9 ng/mL	-	-	[32]
Dengue	1 ng/mL	76	100	[33]
ZIKV	20 ng/mL	81	86	[33]
ZIKV	1 pg/mL	-	-	[34]
ZIKV	45 pg/mL	-	-	[35]
*Coxiella burnetii*	-	92	89	[36]
*Salmonella typhimurium*	3 × 10^3^ CFU/mL	-	-	[37]
*Escherichia coli* O157:H7	10 CFU/mL	-	-	[38]
*Escherichia coli* K-12	3.4 × 10^4^ CFU/mL	-	-	[39]
*Escherichia coli* O157:H7	1.1 × 10^3^ CFU/mL	-	-	[40]
*Escherichia coli* O157:H7	5 CFU/mL	-	-	[41]
*Escherichia coli* BL21	20 CFU/mL	-	-	[41]
*Escherichia coli*	50 CFU/mL	-	-	[42]
*Escherichia coli* O157:H7	100 CFU/mL	-	-	[43]
*Escherichia coli*	10^3^ CFU/mL	-	-	[44]
*P. aeruginosa*	10^3^ CFU/mL	-	-	[44]
